# From Observing Little Animalcules to Detecting Fastidious Bacteria

**DOI:** 10.3201/eid3001.AC3001

**Published:** 2024-01

**Authors:** Byron Breedlove, Clyde Partin

**Affiliations:** Centers for Disease Control and Prevention, Atlanta, Georgia, USA (B. Breedlove);; Emory University School of Medicine, Atlanta (C. Partin)

**Keywords:** Anthonie van Leeuwenhoek, Jan Verkolje, Portrait of Anthonie van Leeuwenhoek, Natural Philosopher and Zoologist in Delft, From spontaneous generation to animalcules to fastidious bacteria, bacteria, bacterial pathogens, microscopes, infectious diseases, fastidious bacteria, antimicrobial resistance, art and science, about the cover, genomic sequencing, biology

**Figure:**
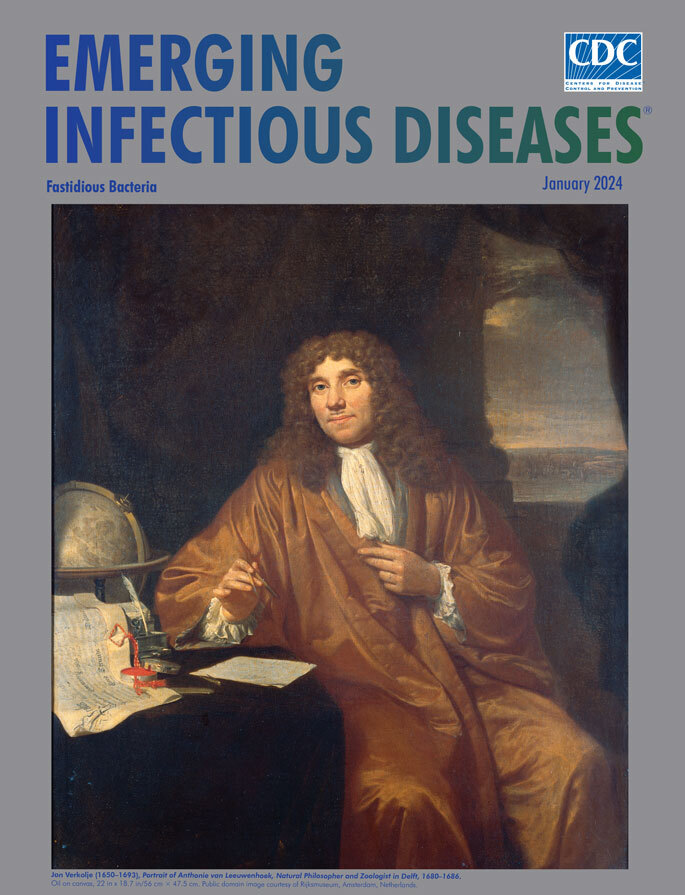
**Jan Verkolje (1650–1693), Portrait of Anthonie van Leeuwenhoek, Natural Philosopher and Zoologist in Delft, 1680–1686.** Oil on canvas oil paint, 22 in x 18.7 in/56 cm × 47.5 cm. Public domain image courtesy of Rijksmuseum, Amsterdam, the Netherlands.

We still think of human disease as the work of an organized, modernized kind of demonology, in which the bacteria are the most visible and centrally placed of our adversaries. We assume that they must somehow relish what they do.

— Lewis Thomas, *The Lives of a Cell: Notes of a Biology Watcher*. Chapter 15: Germs.

Pictured on this month’s cover is a portrait of Anthonie van Leeuwenhoek (sometimes spelled Antony or Antoni), born October 24, 1632, in Delft, the Netherlands, and among the earliest observers of bacteria. A brief biography on the University of California Museum of Paleontology website describes Leeuwenhoek as “an unlikely scientist” who “succeeded in making some of the most important discoveries in the history of biology.” During his younger years, he received no advanced education that would presage his future scientific accomplishments. Leeuwenhoek became a fabric merchant and ran a haberdashery in Delft, where he also held several appointed positions as a surveyor and as a minor city official. At age 16, Leeuwenhoek moved to Amsterdam for 6 years, where, according to infectious disease specialist Robert P. Gaynes, he “became acquainted with Jan Swammerdam, a man known in later years to have fashioned early microscopes.” In the course of his work, Leeuwenhoek used a simple microscope to inspect fabrics used in his drapery and clothing business. 

Although he could not read English, Leeuwenhoek’s interest in microscopy was piqued by *Micrographia* (1665), the popular book by Robert Hooke that detailed and illustrated his microscopic observations. Historian Frank N. Egerton noted that Leeuwenhoek started grinding his own lenses and assembling simple microscopes in 1673 and, during the next 50 years, crafted an estimated 500 microscopes. At the time of Leeuwenhoek’s death, noted Meyer Friedman and Gerald W. Friedland, he had a collection of 247 microscopes and 172 lenses mounted in various precious metals. In 1745, Leeuwenhoek’s daughter Maria auctioned these items, netting 61 pounds. Fewer than 10 of his microscopes are extant. 

The portrait by Dutch artist Jan (Johannes) Verkolje depicts Leeuwenhoek splendidly dressed, wearing a frilled shirt, a fashionably swathed scarf, and an elegant satin robe, perhaps perks of his profession. The text accompanying the painting at the Rijksmuseum states that Leeuwenhoek “is sitting at a writing table on which is a certificate of his appointment as a member of the Royal Society in London by Charles II.” Curiously, no microscopes are featured in this depiction. 

As recounted in *A Brief History of Bacteriology*, Leeuwenhoek was “able to see objects which he called ‘animalcules’ in rain water, and in scrapings from his teeth. He noted that some specimens were motile, and described stick-like shapes and spirals. He did not associate his animalcules with disease. The animalcules have become variously known as germs, microbes, bacteria, micro-organisms or simply ‘organisms.’” Leeuwenhoek’s descriptions represent the primordial steps down a path that eventually proved the role of his “little creatures” to be the etiologic agents of infectious disease. 

Friedman and Friedland also explain that Leeuwenhoek was later puzzled to discover that the plaque on his front teeth had quit harboring his little animals, yet his molars still hosted them. In the interim, he had taken up the habit of drinking scalding coffee, which he realized cleansed his anterior teeth but did not affect his molars. The famous French scientist Louis Pasteur, when he developed his method of pasteurization, would remind us of this theme of utilizing heat as a strategy for managing bacteria.

Eminent scientists continued the arc of Leeuwenhoek’s discovery. In 1678, Robert Hooke, at the request of the Royal Society, confirmed Leeuwenhoek’s “epochal observations.” Leeuwenhoek’s glittering star dimmed after his death in 1723. Four decades after Leeuwenhoek’s death, Austrian Marc von Plenciz resurrected Leeuwenhoek’s investigations and “declared flatly that contagious diseases were caused by the Dutchman’s small animalcules.” The Italian Agostino Bassi “demonstrated experimentally in 1835 that silk worm disease was caused by bacteria.” The anatomist and physician from Bavaria, Friedrich Henle, promoted Leeuwenhoek’s investigations and, as Friedman and Friedland noted, “impressed on his most brilliant student, Robert Koch, the earthshaking implications of Bassi’s work.” Although Pasteur and Koch were frequently at odds with each other, their contributions were the final act in the living drama that connected Leeuwenhoek’s tiny creatures to the unrelenting scourge of infectious diseases. Their work occurred in a lingering milieu of spontaneous generation theory, a concept Leeuwenhoek found ludicrous, and he was incredulous that the idea was still sundering scientific thought. He wrote, “Can there even now be people who still hang on to the ancient belief that living creatures are generated out of corruption?” Pasteur dismissed the notion of spontaneous generation, especially the arguments of Félix Pouchet, as “merely betrayed shoddy lab techniques,” according to medical historian Roy Porter.

More than 350 years after Leeuwenhoek first glimpsed bacteria, a report published in *Lancet* by the Global Burden of Disease 2019 Antimicrobial Resistance Collaborators, which focused on 33 bacterial pathogens and 11 infectious syndromes (excluding tuberculosis), provides global estimates for the horrendous burden of bacterial infections. Key findings include that in 2019, bacterial infections of all types were linked to 7.7 million deaths globally and that after ischemic heart disease, those infections were the second most common cause of death. Deaths from COVID-19 infection add a complex layer to the accounting of deaths associated with infectious disease. The World Health Organization reported, “On 30 January 2020 COVID-19 was declared a Public Health Emergency of International Concern (PHEIC) with an official death toll of 171. By 31 December 2020, this figure stood at 1,813,188. Yet preliminary estimates suggest the total number of global deaths attributable to the COVID-19 pandemic in 2020 is at least 3 million, representing 1.2 million more deaths than officially reported.” 

The 2019 study reported that 5 bacteria caused half of the non–COVID-19 deaths: *Staphylococcus aureus*, *Escherichia coli*, *Streptococcus pneumoniae*, *Klebsiella pneumoniae*, and *Pseudomonas aeruginosa.* Three types of bacterial infections—lower respiratory tract infections, bloodstream infections, and peritoneal and intraabdominal infection—were responsible for more than 75% of the fatalities reported in that study. Despite effective available antibiotics for treating the 33 culprit bacteria identified in that study, the gravest and complex threats to global public health are access to and distribution of those treatments, lack of consistent surveillance and diagnostic abilities, and antimicrobial resistance. 

Traditionally, most bacteria have been propagated on culture plates or other culture media, though some bacteria do not grow well or as quickly by those methods. Recalcitrant bacteria are known collectively as fastidious bacteria,^1^ and they have a predilection to cause, although not limited to, endocarditis. Ensuing infections create a disproportionate amount of human suffering and death. Examples of fastidious bacteria include *Neisseria gonorrhoeae*, *Campylobacter* spp., and *Helicobacter* spp. 

Although Koch and Pasteur did much to develop the germ theory and the cultivation of bacteria in broth and culture media, both significant gifts to science and humanity, fastidious bacteria have continued to present challenges. The problem has been partially solved by recognizing that culturing these organisms requires patience and culture media fortified with certain nutrients. Even those approaches have been supplanted by advanced identification techniques involving genomic sequencing. This issue of EID features articles that describe newer methods for detecting other fastidious bacteria, including *Auritidibacter ignavus*, *Legionella pneumophila*, *Helicobacter fennelliae*, and *Brucella neotomae.* More than 3 centuries later, the foundation laid by Leeuwenhoek steadfastly endures.

## Footnotes

1 The classically recognized fastidious bacteria of clinical concern are known as the HACEK group. The K stands for *Kingella kingae*, named for CDC microbiologist Elizabeth King.
